# Cortical thickness and childhood eating behaviors: differences according to sex and age, and relevance for eating disorders

**DOI:** 10.1007/s40519-024-01675-3

**Published:** 2024-07-19

**Authors:** Edith Breton, Budhachandra Khundrakpam, Seun Jeon, Alan Evans, Linda Booij

**Affiliations:** 1 Department of Fundamental Sciences, Université du Québec à Chicoutimi, Saguenay, Canada; 2 Sainte-Justine Hospital Research Centre, Montreal, Canada; 3 Department of Psychiatry and Addictology, University of Montreal, Montreal, Canada; 4 Montreal Neurological Institute, McGill University, Montreal, Canada; 5 Ludmer Centre for Neuroinformatics and Mental Health, McGill University, Montreal, Canada; 6 Department of Neurology, Yonsei University College of Medicine, Seoul, South Korea; 7 Douglas Mental Health University Institute, Montreal, Canada; 8 Department of Psychiatry, McGill University, Montreal, Canada; 9 Eating Disorders Continuum, Douglas Mental Health University Institute, 6605 Boul. LaSalle, Verdun, H4H1R3 Canada

**Keywords:** Child development, Neuroimaging, Feeding and eating disorders, Sex, Brain cortical thickness, Eating disorder/etiology, Feeding behaviors

## Abstract

**Purpose:**

This study investigated the association between childhood eating behaviors and cortical morphology, in relation to sex and age, in a community sample.

**Methods:**

Neuroimaging data of 71 children (mean age = 9.9 ± 1.4 years; 39 boys/32 girls) were obtained from the Nathan Kline Institute-Rockland Sample. Emotional overeating, food fussiness, and emotional undereating were assessed using the Children’s Eating Behavior Questionnaire. Cortical thickness was obtained at 81,924 vertices covering the entire cortex. Generalized Linear Mixed Models were used for statistical analysis.

**Results:**

There was a significant effect of sex in the association between cortical thickness and emotional overeating (localized at the right postcentral and bilateral superior parietal gyri). Boys with more emotional overeating presented cortical thickening, whereas the opposite was observed in girls (*p* < 0.05). Different patterns of association were identified between food fussiness and cortical thickness (*p* < 0.05). The left rostral middle frontal gyrus displayed a positive correlation with food fussiness from 6 to 8 years, but a negative correlation from 12 to 14 years. Emotional undereating was associated with cortical thickening at the left precuneus, left middle temporal gyrus, and left insula (*p* < 0.05) with no effect of sex or age.

**Conclusions:**

Leveraging on a community sample, findings support distinct patterns of associations between eating behaviors and cortical thickness, depending on sex and age.

## Introduction

Various studies have shown that eating disorders (EDs) have early developmental origins [1–5]. Throughout childhood, maladaptive eating patterns (i.e. overeating, fussy eating and undereating) have been associated with an increased risk of ED symptoms or diagnoses in adolescence [2, 3]. Children with overeating are at risk of binge-eating behaviors in adolescence [3], while those with high levels of fussy eating are at risk of anorexia nervosa (AN)[3]. The preceding suggests that childhood eating patterns might predict ED symptoms development. Yet, neural mechanisms involved in the association between childhood eating behaviors and later-life ED symptoms are unknown.

According to their mother’s reports, 39% of the children in the Quebec Longitudinal Study of Child Development (QLSCD) had exhibited overeating behaviors by age 5 [6]. Further, food fussiness and undereating can be considered as “picky-eating” behaviors [7, 8], which has a prevalence of 22% among preschoolers [7]. Picky-eating eventually fade for most children [7–10]. Little is known about the persistence of picky-eating later in life; however, there is evidence that in some children, picky-eating could be a long-standing behavior with adverse consequences for growth and neurodevelopment [7, 11, 12]. This strengthens the need to better understand such eating behaviors over time, as well as their underlying neurodevelopmental mechanisms.

To date, most studies on brain correlates of childhood eating behaviors concern food choices and preferences [13–15] or overweight and obesity [16–18]. For example, researchers have been using various functional magnetic resonance imaging (fMRI) tasks to assess children’s food preferences, attempting to identify cognitive pathways (e.g., reward response) involved in food choices [13, 17, 19]. One multimodal neuroimaging study using data from the Adolescent Brain Cognitive Development cohort found that differences in brain structure (i.e., surface area, cortical thickness, subcortical volume, fractional anisotropy and mean diffusivity) predicted significant weight gain over a year in children aged 9–10 years [16]. Other studies have found an inverted U-shaped association between body mass index (BMI) and brain gyrification in school-age children [18]. Specifically, both children with low and high BMI had lower brain gyrification [18]. One study examined the association between food-approach eating behaviors during childhood (at 4 or 10 years old) and brain volume at 13 years old, and reported a positive association between the two [20]. However, this earlier study did not consider restrictive eating behaviors, such as fussy eating or undereating behaviors and it did not include brain measurements during childhood.

In addition, sex differences in ED symptoms presentations are well known, with girls and women being considered at higher risk of AN and bulimia nervosa (BN) than boys and men [21–23]. Yet, cultural and social factors such as gendered stereotypes, as well as the lack of inclusion of males in eating disorders research likely have negatively impacted our understanding of eating disorders in boys and men [24]. Still, it is thought that sex differences are less pronounced (and often absent) in binge-eating disorder (BED) and avoidant/restrictive food intake disorder (ARFID) [25]. A study in a Canadian pediatric population found that the clinical presentation of ARFID often differs between sexes, as typically, girls more often present with symptoms related to undereating, whereas boys more often refuse to eat due to sensory characteristics of aliments [26]. Overall, the neurodevelopmental patterns that may help explain the differences and similarities between sexes have not yet been identified.

The aim of the present study was to investigate the link between cortical morphology and child and adolescent eating behaviors that have previously been identified as potential predictors of EDs (i.e., overeating, food fussiness and undereating), in a community-based sample. Furthermore, we studied the moderating role of sex and age in the preceding associations.

## Methods

### Participants

Participants were selected from the Nathan-Kline Institute Rockland sample (NKI-RS) [27]. The cohort was established with the aim of advancing knowledge in psychiatric neuroscience, particularly the developmental factors associated with risk and resilience to mental disorders across the lifespan. As specified in the original study [27], written informed consent and assent were obtained from every child participant and their legal guardian, and the study was approved by the respective Institutional Review Boards and no new data were collected in the context of the present study. The NKI-RS dataset is an open-access database. A data usage agreement was signed prior to the use of the data. Children aged 6 to 15 years were included in this study if complete data on age, sex, eating behaviors were available, and if they had participated in at least one MRI session. For individuals with more than one follow-up, MRI sessions and clinical assessments were at 15-month interval. The sample included scans from *N* = 71 participants, 39 of whom were boys (54.9%), with a mean age of 9.9 ± 1.4 years. A summary of participants’ characteristics can be found in Table 1.

**Table 1:** Participant characteristics

Characteristics	Mean (SD) or *N* (%)
Sex (boys/girls)	39/32 (54.9/45.1)
Mean age (years)	9.9 (1.4), range 6.7–14.9
BMI (kg/m^2^)	18.6 (3.4), range 12.8–31.3
Childhood eating behaviors	
Emotional overeating	1.7 (0.7), range 1.0–4.0
Food fussiness	2.6 (0.9), range 1.0–5.0
Emotional undereating	2.2 (0.7), range 1.0–4.0
MRI scans	
Number of individuals with one scan	31
Number of individuals with two scans	27
Number of individuals with three scans	13

### Measures

#### Children’s Eating Behavior Questionnaire (CEBQ)

Eating behaviors were assessed using the Children’s Eating Behavior Questionnaire (CEBQ) [28], completed by the participant’s primary caregiver. The CEBQ contains 35 items assessing eight eating behaviors. The present study focused on three subscales of the CEBQ: emotional overeating, food fussiness and emotional undereating. This was based on previous studies reporting these behaviors as potential predictors of ED symptoms later in life [2, 3, 5]. Items from each subscale were rated on a 5-point Likert scale from 1 (never) to 5 (always) and a mean was calculated (see Table 2). Previous studies on the psychometric properties of the CEBQ showed Cronbach alpha’s for the different subscales of 0.75 or higher [29].

**Table 2:** Summary of items from the Children’s Eating Behaviors Questionnaire subscales [28]

Subscale	CEBQ Items
Emotional overeating	My child eats more when anxious My child eats more when annoyed My child eats more when worried My child eats more when s/he has nothing else to do
Food fussiness	My child enjoys tasting new foods My child enjoys a wide variety of foods My child is interested in tasting food s/he hasn’t tasted before My child refuses new foods at first My child decides that s/he doesn’t like food, even without tasting it My child is difficult to please with meals
Emotional undereating	My child eats less when s/he is upset My child eats less when s/he is angry My child eats less when s/he is tired My child eats more when s/he is happy

#### Body mass index (BMI)

Study staff measured height (cm) and weight (kg), and BMI was automatically calculated from height and weight during each study visits.

#### Image acquisition, pre-processing and cortical thickness measurements

Information on data acquisition can be find elsewhere (http://fcon-1000.projects.nitrc.org/indi/enhanced/mri-protocol.html; [27]). Structural MRI data were pre-processed with the CIVET pipeline (http://www.bic.mni.mcgill.ca/ServicesSoftware/CIVET), and cortical thickness measurements were obtained at 81,924 vertices covering the entire cortex. The T1-weighted image were first corrected for non-uniformity, and linearly registered to a standard space (Talairach-like MNI152 template, established from the ICBM152 dataset). Brain tissues were segmented into grey matter (GM), white matter (WM), and cerebrospinal fluid based on non-linear registration and the artificial neural network using priors defined in the MNI152 template. Inner and outer GM surfaces were extracted using the Constrained Laplacian-based Automated Segmentation with Proximities (CLASP) algorithm. Then, the Laplacian distance between the two surfaces at 81,924 vertices were used to measure cortical thickness. To force a normal distribution on the corticometric data and to increase the signal to noise ratio, each individual’s map of cortical thickness was blurred using a 30-mm full width at half maximum surface-based diffusion smoothing kernel.

Quality control (QC) of the pre-processed data was performed by two independent experts with scores (0 = failed, 1 = questionable, 2 = passed), and only the scans with consensus were included. QC steps included exclusion of data with low signal to noise ratio, motion artifacts, artifacts due to hyperintensities from blood vessels, surface-surface intersections or poor placement of the GM or WM surface. In total, 530 MRI scans from 162 participants were downloaded from the NKI-RS website (**htttp***://fcon_1000.projects.nitrc.org/indi/enhanced/). Of these, 85 scans failed the QC process and 150 scans were questionable, resulting in 380 scans from 141 participants (see NKI_QC at https://github.com/bkhundrakpam/Project-on-Childhood-Eating-Behaviours). Filtering for CEBQ scores resulted in the final study sample of *N* = 155 scans from 71 participants (Table 1).

### Statistical analyses

Data were analyzed using mixed effect generalized linear models (GLM)s at vertex-level (79,950 vertices covering the entire cortex without ventricule region) and were quantified using *t*-statistics. Significant associations were assessed with multiple comparisons corrected *p*-statistic using random field theory (RFT) [30]. Statistical analyses were conducted using MATLAB (R2019b).

First, the associations between cortical thickness and the emotional overeating, food fussiness and emotional undereating subscales of the CEBQ were assessed separately. For each three CEBQ subscales, cortical thickness was included as outcome and one CEBQ subscale, age, sex, and an age*sex interaction were included as predictors. Participant was added as a random factor in the GLM models to control for having some participants with one, two or three measurement times.

To investigate sex-specific patterns in the association between eating behaviors and cortical thickness, the models were re-estimated separately for boys and girls. Re-estimation was only calculated for the CEBQ subscales showing an interaction of sex in step one.

To clarify the role of age in the association between childhood eating behaviors and cortical thickness, an age-centered approach was used, again only for the CEBQ subscales displaying an interaction of age in step one. An age-centered approach has previously been used for investigating age-specific associations between behavior and brain measures [31, 32]. In this approach, linear mixed models were computed at different centered ages. For example, for age 10 years, 10 was subtracted from the age at data acquisition and this value was put in as the age term. Then, the GLM computed the effect of CEBQ scores on cortical thickness at each age based on values estimated from developmental curves modelled on all data.

Finally, because BMI is typically related to eating behaviors [6, 33, 34], a sensitivity analysis including BMI allowed us to assess its contribution to the preceding associations.

## Results

### Emotional overeating

**Fig. 1:**
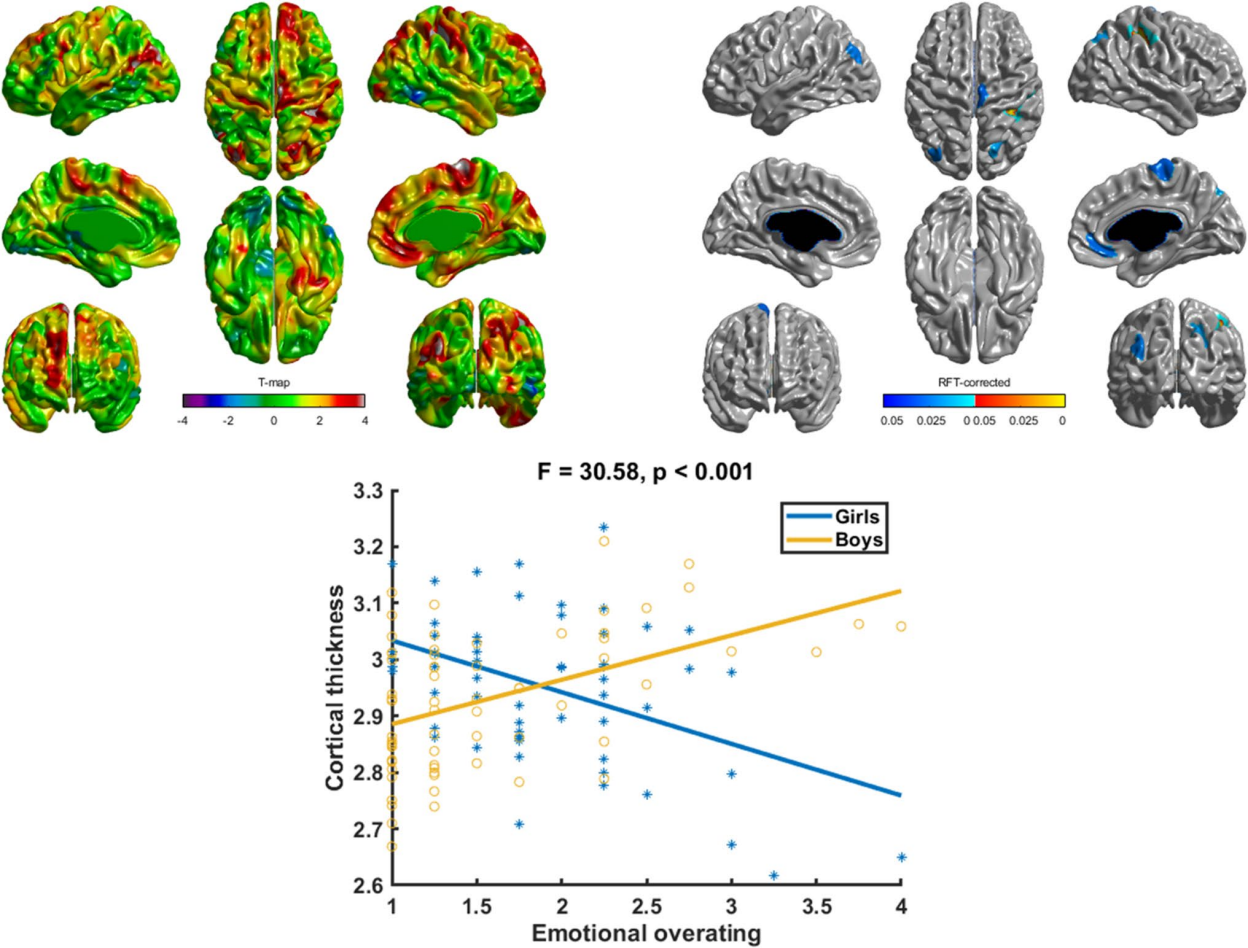
Interaction of sex and emotional overeating on cortical thickness. The interaction of sex and emotional overeating scores on cortical thickness is represented by *t*-statistics (left), while the right panel shows the cortical regions with significant interaction (*p* < 0.05). At the peak vertex of interaction, scatter plots are shown separately for boys and girls. Notably, girls showed thinning with increasing emotional overeating while boys showed thickening with increasing emotional overeating

Table 3 presents the associations between cortical thickness and the emotional overeating, food fussiness and emotional undereating subscales of the CEBQ. Whereas there was no overall association between cortical thickness and emotional overeating, there was a moderating role of sex in the association. In girls, cortical thickness in the right postcentral and bilateral superior parietal gyri was negatively associated with emotional overeating (*p* < 0.05, RFT corrected; Fig. 1). Thus, girls with more emotional overeating had a thinner cortex in these regions. The preceding associations were in the opposite direction for boys (*p* < 0.05, RFT corrected; Fig. 1), indicating that boys with more emotional overeating had a thicker cortex in these brain regions. No age effect was observed for the association between emotional overeating and cortical thickness.

**Table 3:** Association of cortical thickness and childhood eating behaviors (i.e., emotional overeating, food fussiness and emotional undereating)

	Statistically significant correlation (Y/N)^a^, direction ( ±)	Brain region
Emotional overeating	N	NA
Food fussiness	Y (−)	Right inferior parietal
Emotional undereating	Y ( +)	Left precuneus, left middle temporal, left insula

*N*, 125 scans; Y, yes; N, no; NA, not applicable

^a^RFT-corrected

### Food fussiness

**Fig. 2:**
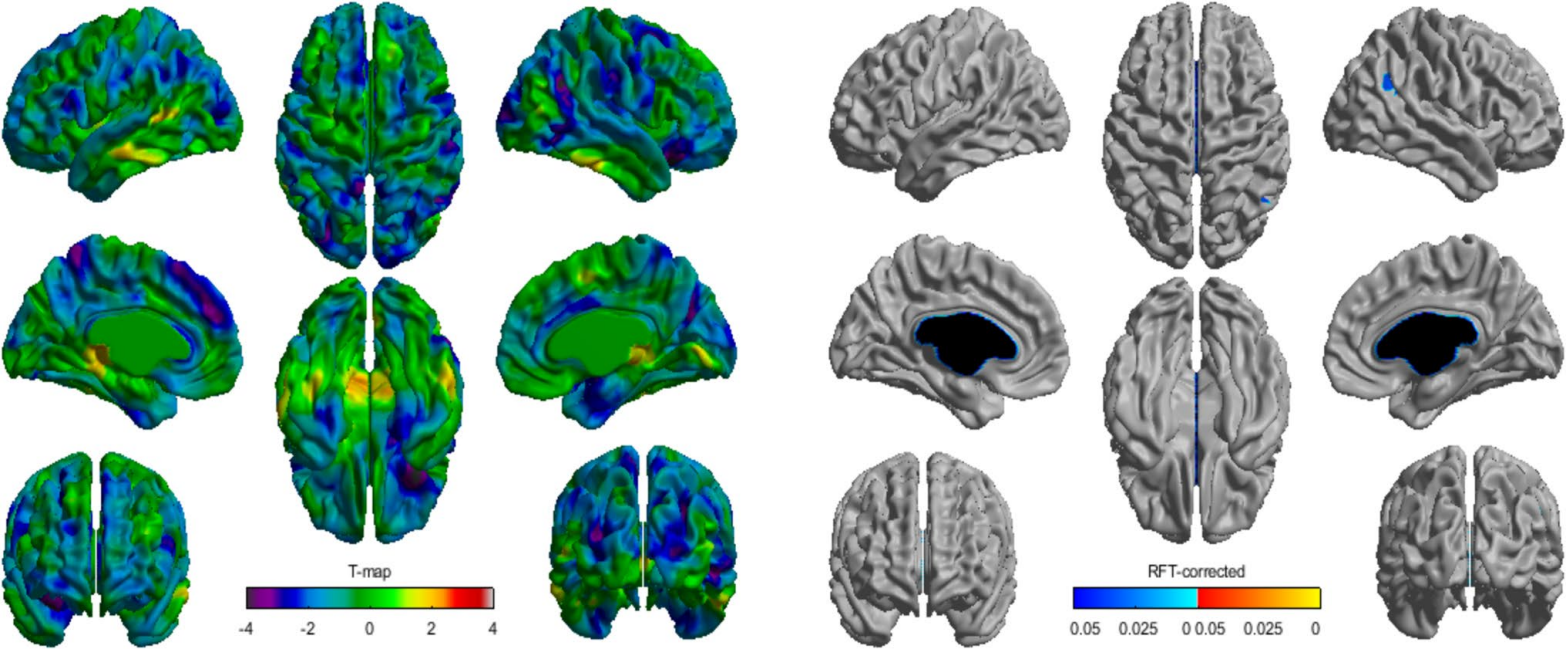
Association of food fussiness and cortical thickness. The association of food fussiness and cortical thickness is represented by *t*-statistics (left), while the right panel shows the cortical regions with significant interaction (*p* < 0.05). We observed significant thinning with food fussiness localized at the right inferior parietal cortex

**Fig. 3:**
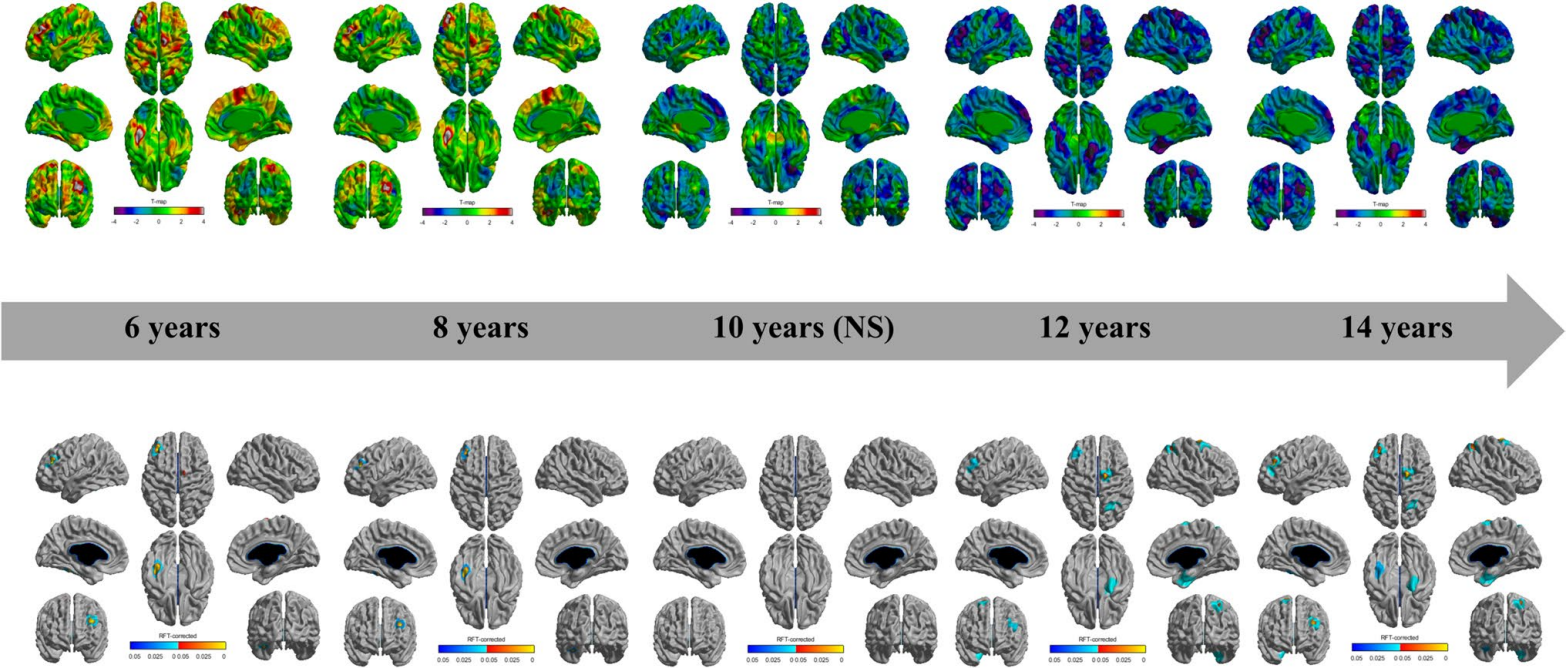
Association of food fussiness and cortical thickness with age using age-centering approach. The association of food fussiness and cortical thickness with age is represented by *t*-statistics (upper panel), while the lower panel shows the cortical regions with significant association (*p* < 0.05). During childhood (6 years and 8 years), we observed significant positive associations between food fussiness and cortical thickness, while during adolescence (12 and 14 years), we observed significant negative associations between food fussiness and cortical thickness

There was a negative association between cortical thickness and food fussiness in the right inferior parietal gyrus, suggesting that children with more fussy eating showed cortical thinning in this brain region (*p* < 0.05, RFT corrected; Fig. 2). Further, an age-centered approach highlighted different patterns of association between food fussiness and cortical thickness according to age (*p* < 0.05, RFT corrected; Fig. 3). The left rostral middle frontal gyrus displayed a significant positive correlation with food fussiness from 6 until 8 years (i.e., thickening in children with more fussy eating), and there was no significant association at 10 years old. Notably, between 12 and 14 years, the association was negative, indicating that young adolescents who had more fussy eating presented cortical thinning.

### Emotional undereating

**Fig. 4:**
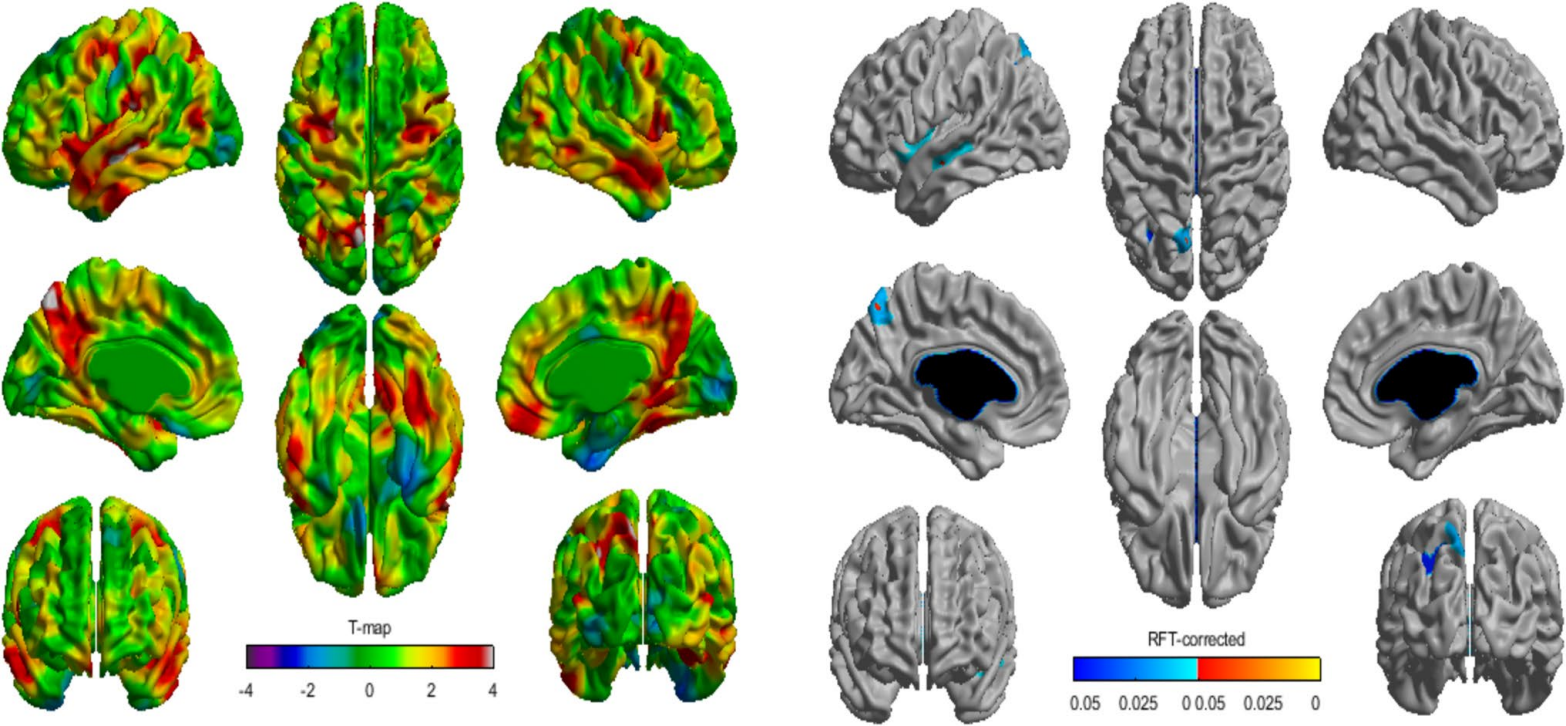
Association of emotional undereating and cortical thickness. The association of emotional overeating and cortical thickness is represented by *t*-statistics (left), while the right panel shows the cortical regions with significant interaction (*p* < 0.05). We observed significant thickening with emotional overeating localized at the left precuneus, the left insula and the left superior temporal gyrus

Children with more emotional undereating had a greater cortical thickness in the left precuneus, left middle temporal gyrus and left insula (*p* < 0.05, RFT corrected; Fig. 4). No interaction of sex or age was observed on this subscale.

### Sensitivity analyses

Including BMI in the models did not change interpretation (i.e., effect size/direction of effect) of the preceding findings.

## Discussion

The present study reported an association between eating behaviors and cortical thickness in a community-based sample of children aged 6 to 15 years. The association between cortical morphology partly varied according to sex and age.

Our results suggest that the association between brain structure and eating patterns might already be, at least in part, established early in development. However, it remains unknown whether this association reflects a differential risk, or a consequence of maladaptive eating patterns. To date, developmental studies support the association between early eating behaviors and disordered eating in adolescence [3, 5], and brain differences have been identified in EDs [35, 36]. The current study contributes to the literature because most existing studies to date have focused on the link between brain processes and BMI [16–18] or food preferences/food choices [13–15, 17], but not on specific eating patterns linked to ED symptoms across development.

A novel finding from the present study is the identification of sex differences in the association between cortical morphology and emotional overeating. Girls presented cortical thinning with increasing emotional overeating, while boys showed the opposite. The direction of effects in girls in the right postcentral and bilateral superior parietal gyri is consistent with some studies in women with BN showing cortical thinning in similar brain regions [37–39]. Findings related to emotional overeating are also in line with evidence of sex differences in children’s food choices and preferences [8]. Parental practices and differences in attitude toward girls and boys could play a role in these sex differences [8]. In the future, it would be of interest to consider gendered factors related to eating behaviors and brain processes, given that gender has been associated with differences in the clinical presentation of EDs [24, 40, 41].

Uncontrolled eating (an umbrella term for a few psychological constructs related to loss-of-control overeating and emotional eating) has been associated with EDs [42–44], and particularly EDs involving binge-eating symptoms [42]. Uncontrolled eating has been associated with temperament traits (e.g., self-directedness) and cognitive processes (e.g., reward sensitivity, cognitive control) that have been linked to EDs [43, 44]. The present study complemented previous studies by identifying brain correlates of emotional overeating in childhood, which may be relevant to the development of EDs later in life.

Children with fussy eating displayed cortical thinning in the right inferior parietal gyrus, and those with undereating behaviors presented cortical thickening in the left precuneus, left middle temporal gyrus and left insula. Both behaviors are components of picky-eating, which can be considered a precursor to ARFID [25]. The lack of sex interaction between cortical thickness and the food fussiness and emotional undereating subscales is consistent with lack of pronounced sex differences typically reported in picky-eating and ARFID [26, 45].

Notably, the age-centered approach revealed a dynamic pattern of association between food fussiness and cortical morphology. The region of the left rostral middle frontal gyrus was thicker in younger children with high levels of food fussiness but thinner in older children with high levels of food fussiness. This finding is of interest in relation to previous research on ARFID and neurodevelopmental disorders. Given that most children grow out of picky-eating [7–10], it is possible that the persistence of picky-eating behaviors might be related to an atypical neurodevelopmental pattern. Further, the neurodevelopmental trajectory of children who continue to exhibit food fussiness in adolescence may, in part, be similar to what is observed in youth with some neurodevelopmental disorders. Supporting this hypothesis, neurodevelopmental disorders are frequently comorbid with ARFID [25, 45–48], and autism spectrum disorder has been associated with the chronicity of problematic eating patterns in children [45, 49]. In the present study, we found a reduction in cortical thickness in the left rostral middle frontal gyrus in children who continued to exhibit food fussiness in adolescence. This cortical thinning was located in a brain region previously highlighted in individuals with neurodevelopmental disorders, including autism spectrum disorder, attention deficit/hyperactivity disorder and schizophrenia [50, 51]. These results suggest that there may be a neurobiological link between certain maladaptive childhood eating behaviors and neurodevelopmental disorders, although more work is needed to test this hypothesis.

Lastly, the positive association between emotional undereating and cortical thickness in the left precuneus, left middle temporal gyrus and left insula is in line with previous studies on brain correlates of restrictive eating behaviors in women [52, 53]. These brain regions have been related to emotion perception and social cognition [52], two important cognitive aspects in EDs. Our results indicate that at least some neural differences might be observable during childhood in individuals with undereating behaviors. Overall, future studies should consider brain processes and eating behaviors from childhood onward to determine whether neurobiological differences could be involved in the transition from early maladaptive eating patterns to long-term risk of EDs, as few (if any) studies have investigated the link between early eating behaviors, neurodevelopment and risk of EDs altogether.

The strengths of the current study include the inclusion of both boys and girls, and the use of a standardized imaging protocol in a well-characterized sample. Still, results should be considered in light of some limitations. The CEBQ is a parent-reported questionnaire, which can induce a social desirability bias. Also, the number of brain scans was not the same for all individuals (which was controlled for by adding participants as a random factor in the statistical analyses) and the sample size was relatively small, possibly limiting statistical power.

In conclusion, the present study provides evidence for an association between cortical morphology and childhood eating behaviors. We found that sex was a moderator of the association between cortical thickness and emotional eating, while age moderated the association between cortical thickness and food fussiness. Findings may have implications for the early detection and prevention of EDs, as they may contribute to the understanding of early brain differences associated with childhood eating behaviors, which may be present before the development of full-blown EDs and could represent behavioral and neurobiological risk factors.

## Data Availability

Scripts are available at https://github.com/bkhundrakpam/Project-on-Childhood-Eating-Behaviours
